# Protective Effect of Whey Protein and Polysaccharide Complexes on *Lactobacillus paracasei* F50: Comparative Analysis of Powder Characteristics and Stability

**DOI:** 10.3390/foods14091555

**Published:** 2025-04-28

**Authors:** Xinrui Zhang, Xiaowei Peng, Huijing Chen, Aijun Li, Gang Yang, Jianquan Kan

**Affiliations:** College of Food Science, Southwest University, Chongqing 400715, China; 19562244580@163.com (X.Z.); qqajx19@163.com (X.P.); chj1451320167@163.com (H.C.); liaiijun2021@163.com (A.L.); yanggangresearch@163.com (G.Y.)

**Keywords:** whey protein, κ-carrageenan, *Lactobacillus paracasei* F50, spray drying

## Abstract

To enhance *Lactobacillus paracei* F50 viability during spray drying and long-term storage, this study evaluates whey protein (WP) crosslinked with four polysaccharides (κ-carrageenan (KC), xanthan gum (XG), low-methoxyl pectin (LMP), sodium alginate (SA)) for the first time as protective matrices for *L. paracasei* F50 during spray drying. The four kinds of crosslinked wall materials were compared by various characterization methods. Among them, the WP-κ-carrageenan (WP-KC) composite exhibited optimal performance, forming a uniform microcapsule with high colloidal stability. After spray drying, WP-KC achieved the highest viable cell density (9.62 lg CFU/g) and survival rate (91.85%). Notably, WP-KC maintained viability above 8.68 lg CFU/g after 120 days of storage at 4 °C, surpassing other formulations. Structural analysis showed that the WP-KC microcapsule was completely encapsulated without breaking or leaking and confirmed the molecular interaction between WP and KC. Under the condition of high temperatures (≤142.63 °C), the wall material of the microcapsule does not undergo any endothermic or exothermic process and is in a state of thermodynamic equilibrium, with excellent stability and good dispersion. Additionally, microcapsules exhibited enhanced resistance to thermal stress (55–75 °C) and UV irradiation, higher than that of free cells. These results highlight WP-KC as an industrially viable encapsulation system for improving probiotic stability in functional foods, offering critical insights into polysaccharide–protein interactions for optimized delivery systems.

## 1. Introduction

Probiotics, a class of active microorganisms that are beneficial to the host, have been shown to enhance human intestinal health and cellular immunity. *Lactobacillus paracasei* is a probiotic commonly used in human healthcare and food development. Research has shown that *L. paracasei* can play a variety of health roles, such as regulating the intestinal flora to prevent obesity [1] and gastric cancer [2]. To use probiotics for subsequent healthcare and biocontrol, probiotic liquids need to be transferred from the laboratory for commercialization [3]. For example, liquid bacterial solutions embedded in hydrogels, emulsions, and nanocoatings can only be used for the short-term embedding of lactic acid bacteria and cannot be stably stored and applied for a long time [4]. Therefore, it is particularly important to make microcapsule powders convenient for storage and transportation. During spray drying without additional protection, *L. paracasei* suffers from heat stress, in which damage to the cell membrane and ribosome is the key factor influencing heat death [5], and the leakage of wall materials during storage affects the activity of *L. paracasei*. In summary, *L. paracasei* is widely used in food, and its efficient utilization and stable storage are important.

As *L. paracasei* is unstable during storage, and microcapsules have the functions of sustained release and extending shelf life, the application of microencapsulation technology can prolong the survival time of the strain. Some synthetic polymer materials (such as polyvinyl alcohol) have poor biodegradability as microcapsule wall materials, which may lead to environmental accumulation or long-term residues in the human body, and there are chronic toxicity risks. Considering food safety issues, proteins and polysaccharides are the best choices. In recent years, protein–polysaccharide complexes have garnered significant attention owing to their ability to form electrostatic interactions between oppositely charged groups under specific conditions. These interactions drive composite coacervation, enabling the effective encapsulation of bioactive substances. For instance, in one study, gelatin and gum arabic were employed to microencapsulate *Lactobacillus plantarum* via dual emulsification and coacervation, achieving 90% cell viability after 45 days of storage at 8 °C and −18 °C [6]. Interactions between proteins and polysaccharides occur via two mechanisms: covalent and non-covalent. But they are significantly modified when they are treated with high pressure, ultraviolet radiation, and temperature or pH adjustment, which affect their functional properties, including solubility, viscosity, emulsifying ability, and gelling ability, as well as structural properties such as conformational change and denaturation [7]. Studies have shown that the addition of three polysaccharides to WPI to form a complex enhances its binding to curcumin [8]. Among these polysaccharides, λ-carrageenan (λ-CG), a sulfated polymer with exceptional gelling, thickening, and film-forming properties, is widely utilized in food systems. Studies reveal that increasing λ-CG content strengthens its interaction with soy protein isolate via electrostatic and hydrogen bonding, improving emulsion stability [9]. Whey protein (WP), a milk-derived, nutrient-rich protein, exhibits hydrophobic domain exposure during spray drying, facilitating adhesion to lactic acid bacteria and enhancing probiotic survival rates [10].

Although protein–polysaccharide complexes demonstrate potential for bioactive protection, critical gaps persist in probiotic encapsulation. Polysaccharide molecular properties—such as charge density and chain rigidity—directly modulate synergies with WP. For example, low-methyl pectin forms pH-sensitive electrostatic complexes with WP encapsulating *Lactobacillus rhamnosus* in W/O/W emulsions at pH 3, yet acidic conditions risk bacterial stress [11]. Sodium alginate’s linear stiffness generates porous microcapsules during spray drying, whereby rapid shell solidification restricts bubble expansion, leading to structural collapse [12]. Xanthan gum enhances thermal stability for *Lactobacillus casei* but limits hydrophobic domain exposure during drying [13]. In contrast, κ-carrageenan uniquely strengthens WP systems: its sulfate groups enable robust electrostatic crosslinking near WP’s isoelectric point (pH 4.5–5.2), forming thermally stable 3D networks that resist spray-drying phase separation and stabilize bacterial membranes via hydrogen bonding. Unlike other carrageenans, κ-CG promotes WP aggregate reorganization into mechanically robust fractal particles at 75 °C without protein loss [14]. However, systematic comparisons of κ-CG with other polysaccharides in WP complexes are lacking, particularly regarding protective mechanisms for oxygen-sensitive, thin-walled probiotics such as *Lactobacillus paracei*.

In this study, we compared the protective effects of microcapsules composed of whey protein and four polysaccharides (κ-carrageenan (KC), low methoxyl pectin (LMP), xanthan gum (XG), and sodium alginate (SA)) on *L. paracasei* F50. In addition, the four types of microcapsule powders prepared by spray drying were characterized using scanning electron microscopy (SEM), infrared spectroscopy (IR), differential scanning calorimetry (DSC), and X-ray diffraction (XRD). Particle size and zeta potential were also measured. This study aimed to increase the storage stability of *L. paracasei* powder and reveal methods to cope with adverse conditions during food processing. This study not only provides an effective method for the microencapsulation of *Lactobacillus paracaseus* F50 but also explores and compares the microcapsule wall structure formed by WP and KC, LMP, XG, and SA.

## 2. Materials and Methods

### 2.1. Materials

Whey protein (purity ≥ 90%, EINECS number 207-683-6) was obtained from Hefei Bomei company (Hefei, China); κ-carrageenan (purity ≥ 98%, CAS number 11114–20-8, Lot number A2418057) from Aladdin biochemical technology (Shanghai, China); sodium alginate (purity ≥ 90%, CAS number 9005-38-3, Lot number S817372, M/G=1:1200 ± 20 mPa.s) from Shanghai McLean Biochemical Technology Co., Ltd. (Shanghai, China); xanthan gum (purity ≥ 98%CAS number 11138-66-2, Lot number JS253184) from Shanghai Yuanye Biotechnology Co., Ltd. (Shanghai, China); and low-methoxyl pectin (degree of esterification ≤ 25.0%, CAS number 9000-69-5) from Hefei Bomei company (Hefei, China). *L. paracasei* F50 was isolated and identified by our research team and stored at the China Type Culture Collection Center (Wuhan, China) under the accession number CCTCC No: M 20222039. All the other chemical reagents used were of analytical grade.

### 2.2. Preparation of L. paracasei F50 Suspension

*L. paracasei* F50, stored at −20 °C in glycerol tubes, was activated in de Man–Rogosa–Sharpe (MRS) liquid medium and cultured at 37 °C until the end of the logarithmic period (final cell concentration: 1.7 × 10^10^ CFU/mL) [15]. The bacterial liquid was collected during the stable period, and 45 mL of the *L. paracasei* fermentation liquid was centrifuged at 2000×g for 8 minutes at 4 °C. The supernatant medium was discarded, the bacterial mud sediment was retained, and 0.85% normal saline for washing was added. This was repeated twice, and then it was resuspended in 5 mL of normal saline and shaken well for use.

### 2.3. Preparation of Microcapsule Wall Material

κ-carrageenan, xanthan gum, sodium alginate, and low-methoxyl pectin were selected as polysaccharide components to prepare 0.5% (*w/v*) κ-carrageenan solution, 0.5% (*w/v*) xanthan gum, 1% (*w/v*) low-methoxyl pectin, and 1% (*w/v)* sodium alginate. The method described by Sharifi was used [16]. In total, 40 mL of 3% (*w/v*) whey protein solution was prepared, 15 mL of the bacterial mud suspension prepared in Section 2.2 was added, and it was mixed well according to a ratio of protein:polysaccharide = 2:5. In total, 40 mL of whey protein was slowly added into the four 100 mL polysaccharide solutions described above. Homogenization was performed for 1 min, and 1% (*w/v*) lactic acid was added to stimulate the electrostatic binding between the whey protein and the polysaccharides. The pH of the final solution was adjusted to 4 according to the best complex condensation results obtained in the pre-experiment. Then, 10 mL of the thickening stabilizer sodium carboxymethyl cellulose and 100 mL of 10% (*w/v*) β-cyclodextrin were added. The liquid microcapsules were prepared by stirring at 600 rpm and then kept at 4 °C for 12 h to ensure the formation of complex condensates.

### 2.4. Preparation of Microcapsules

**Figure 1:**
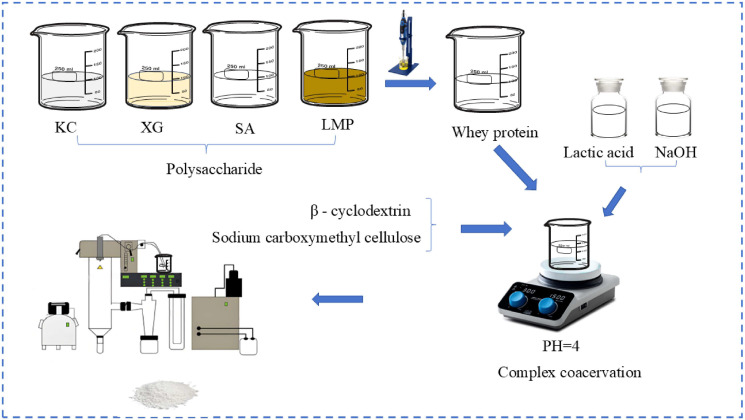
Preparation of microcapsules by spray drying.

Microcapsules were prepared (Figure 1) by spray drying the mixture of bacterial suspension and wall material. Briefly, spray drying was performed using a laboratory spray dryer (B-290, Büchi, Switzerland) with a 1.5 mm diameter atomizing nozzle [17]. The operating conditions, determined by referring to other literature studies and the pre-experimental research of this experiment, were a Feed rate between 20 and 30 mL/min, an inlet temperature of 110 °C, an outlet temperature of 60 °C, and an atomizing air flux of 0.15 MPa.

### 2.5. Characterization of Microcapsules

#### 2.5.1. Cell Survival

To determine the number of viable bacterial cells in the microcapsules, 0.2 g of the prepared microcapsule powder was weighed and dissolved in 5 mL of sterile normal saline. Subsequently, the suspension was spread evenly on an MRS agar plate. After incubation at 37 °C for 48 h, the strain density was observed and recorded. Three parallel experiments were performed. The bacterial survival rate was calculated using Formula (1), as follows:

(1) $$\mathrm{Survival}\;\mathrm{rate}\;(\%)=\frac{N}{N_{0}}\times 100$$

where *N* is the number of viable cells per unit volume of sample after drying (in CFU/g), and *N*_0_ is the number of viable cells per unit volume of sample before drying (in CFU/g).

#### 2.5.2. Physical Properties

The moisture content of the microcapsules was calculated from the decrease in the dry weight [18]. The hygroscopicity was evaluated by measuring the weight gain of 1g of microcapsule powder after storage for 7 days at 25 °C and 75.3% RH [19]. The microcapsule powder (2 g) was dissolved in 50 mL of distilled water, and the time required for complete dissolution and disappearance of the probiotic microcapsule powder was used to indicate solubility. The Hausner ratio (*HR*) is an important parameter used to evaluate the flowability of powders [20] and reflects the density changes of powders between the loose and compacted states. The Carr Index (*CI*) was used to describe the flow properties of the powders in air. The *CI* affects the flowability of powders in air and can be easily transported through pipelines. Each sample was measured three times for each index. *HR* (2) and *CI* (3) were calculated using the following formula:

(2) $$HR=\frac{\rho T}{\rho L}$$

(3) $$CI(\%)=\frac{\rho T-\rho L}{\rho T}\times 100$$

where *ρT* represents tap density, and *ρL* represents bulk density.

#### 2.5.3. Morphological Analysis

A SEM (Phenom Pro, Phenom world, Holland) was used to observe the apparent morphological characteristics and particle size of the microcapsule powder. After spray drying, the microcapsules were placed on double-sided carbon tape adhered to an aluminum support and then sprayed with gold by ion sputtering under vacuum conditions. At an accelerating voltage of 20 kV, samples at different magnifications were scanned and photographed using a secondary electron detector.

#### 2.5.4. Particle Size and Zeta Potential Analysis

In total, 0.2 g of the spray-dried microcapsule powder was weighed and dissolved in 5 mL of distilled water. The suspension was incubated in a shaker at 37 °C for 30 min. The particle size distribution, zeta potential, and the polydispersity index (PDI) of the suspension were determined using a laser particle size analyzer (Nano ZS90, Malvern, UK). Each sample was measured three times for each index.

#### 2.5.5. Differential Scanning Calorimetric Analysis of Thermal Stability

The measurements were conducted using a differential scanning calorimeter (DSC 4000, PerkinElmer, Shelton, CT, USA). In total, 10 mg of microcapsule powder was placed in an aluminum pan, with the heating rate set at 10 °C/min [21]. The glass transition temperature and melting temperature of various materials were assessed over a temperature range from room temperature to 200 °C, utilizing an empty aluminum pan as the reference.

#### 2.5.6. Infrared Spectroscopy Analysis

An IR spectrometer (Tracer-100, Shimadzu, Kyoto, Japan) was used to determine the crosslinking effect between the various materials. The sample underwent 16 scans between wavelengths of 4000 and 400 cm^−1^. Gaussian fitting of broad peaks in the range of 3000–3800 cm^−1^ was used to obtain the proportion and distribution of various types of hydrogen bonds in the sample.

#### 2.5.7. Crystal Structure Analysis

The crystal structure distribution of the microencapsulated powder was determined using an X-ray diffractometer (X’Pert3, PANalytical, Almelo, The Netherlands). The working voltage was 40 kV, the copper target current was 40 mA, and Cu Kα was used as the diffraction source. The diffraction patterns were recorded in the range of 5 to 90° (2θ), with a scanning speed of 2°/min [22].

### 2.6. Determination of WP-KC Microcapsule Properties

#### 2.6.1. The Storage Stability of the Microcapsules

The microcapsule powder was stored for 120 days at −20, 4, and 25 °C. Every 15 days, 0.2 g of the microcapsule powder was taken, dissolved in 5 mL of sterile normal saline, and then cultured on MRS plates for 2 days, and the strains were observed and counted [23]. Three parallel experiments were performed.

#### 2.6.2. Growth Curve and Acid Production Capacity

Strains before and after microencapsulation were inoculated in MRS liquid medium for 48 h. Samples were collected every 8 h, and the UV absorbance at a wavelength of 600 nm was measured to construct the growth curve of the strains. Following dilution of the bacterial solution, a 0.4% (*w/v*) FeCl_3_ solution was added and thoroughly mixed. The UV absorbance value at a wavelength of 390 nm was then determined, allowing for the construction of the acid production curve. Three parallel experiments were performed.

### 2.7. Effects of Food Processing Conditions on Microcapsules

#### 2.7.1. Thermal Stability

In total, 0.2 g of microcapsules and an equal density of strain suspension were added into the aseptic tubes, and 4.8 mL of phosphate-buffered saline was added for water bath heating. The heating disinfection method based on the food industry as maintained for 10 min at 55 °C, 65 °C, and 75 °C. Then, the samples were cultured on the MRS plates for 2 days, observed, and counted. Three parallel experiments were conducted.

#### 2.7.2. Ultraviolet Radiation

In total, 0.2 g of microcapsules and an equal density of strain suspensions were added to sterile tubes, which were both exposed to 235.7 nm UV for 1 h and protected from light for 30 min. Subsequently, the samples were transferred into sterile tubes containing phosphate-buffered saline, and cell counts were performed according to the aforementioned method. Three parallel experiments were performed.

### 2.8. Statistical Analysis

All experiments in this study were repeated three times, and parallel data are expressed as the mean ± standard deviation. All data analyses and processing were completed using Origin 2018 and SPSS 22. Dunn’s test was used to compare differences between groups. Differences with a *p* value of less than 0.05 were considered statistically significant. The graphs were drawn using Origin 2021 and PowerPoint 2020.

## 3. Results and Discussion

### 3.1. Strain Density and Survival Rate of Microcapsules

**Figure 2:**
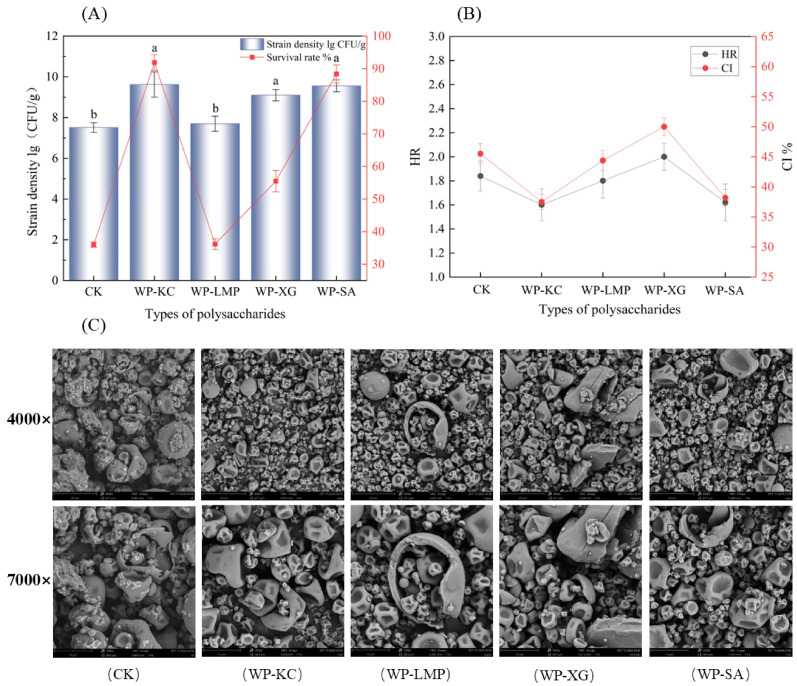
Density and survival rate of microcapsules containing whey protein and different polysaccharides (**A**). Hausner ratio and Carr Index of microcapsule powder with different wall materials (**B**). SEM image of microcapsules (**C**). Note: The letters a, b in (**A**) indicate significant differences in results in the same column.

The powder obtained by spray drying after crosslinking the complex condensation of whey protein and the four polysaccharides is shown in Figure 2A for strain density and survival rate. The WP-KC group, the maximum density of bacteria was 9.62 ± 0.62 lg colony-forming units per gram (CFU/g; the same below) (*p* < 0.05), and the survival rate of bacteria was 91.85 ± 2.42%. The second highest was WP-SA at 9.56 ± 0.29 lg CFU/g (*p* < 0.05), and the survival rate was 88.4 ± 2.76%. Studies have revealed that the stable double-network emulsion gel formed by WP and KC can be used to enhance the vitality of probiotics during gastrointestinal digestion and the hot processing of foods [24]. The complex coacervates exhibit superior viscoelastic properties, likely due to a more densely packed and mechanically stable microstructural network [25].

### 3.2. Moisture Content, Solubility, Hygroscopicity, Hausner Ratio (HR), and Carr Index (CI)

The moisture content has a significant effect on the storage stability, fluidity, and solubility of the powder. Excessive moisture content leads to powder deterioration, caking, or reduced fluidity during storage. The lower the moisture content is, the higher the storage stability of the bacterial powder. However, an excessively low moisture content can also adversely affect active substances [26]. As shown in Table 1, the moisture content of the WP-LMP group (2.35 ± 0.02%) was significantly higher than that of the other groups (*p* < 0.05), and the other groups all maintained appropriate and similar water content (*p* > 0.05). These results indicate that pectin can retain residual water during spray drying. In contrast, wall materials such as WP-KC and WP-XG create a stable microenvironment by limiting water adsorption and maintaining the glass transition temperature, thereby improving storage stability. The solubility showed that WP-SA (300 ± 15.36 s) formed a dense network structure soon after dissolution. However, in the CK group (900 ± 20.53 s), because β-cyclodextrin could not be completely dissolved in cold water, the undissolved part existed in the form of precipitation, could not form a uniform and stable medium, and was not easily dissolved, and the dissolution time was the longest.

It is worth noting that when compared with CK, the moisture absorption of all wall materials was significantly reduced (2.08 ± 0.24 g/100 g) (*p* < 0.01). Among the wall materials, WP-KC (780 ± 13.42 s) and WP-XG (660 ± 12.84 s) exhibited moderate solubility, equilibrium protection, and targeted release. This is due to the hydrophilic interaction of sulfate or carboxyl groups, which effectively prevents water penetration. These results showed that the water content and moisture absorption of the four powders were lower than those of other microencapsulated powders [27] of the same type and had better stability, highlighting WP-CK and WP-XG as promising candidates for *L. paracei* F50 protection.

HR and CI values can explain the flowability of microcapsule powders [28]. For dry powders, the lower the HR and CI values are, the better the flowability of the powder. Powders with good fluidity can maintain uniform dispersibility, thereby reducing physical stress-induced damage to the microcapsules, and maintaining the integrity of the embedded layer to delay the infiltration of water and oxygen, thus reducing the influence of external environmental fluctuations on bacteria and the decay rate of viable bacteria during storage [29]. Figure 2B shows the flow characteristics of microcapsules with different protective wall materials under spray drying. After comparing the four protective wall materials, it was found that when WP-KC was used as the wall material, the HR and CI values were the smallest, which were 1.6 ± 0.13 and 37.5 ± 1.25% (*p* < 0.05), respectively. WP-SA followed, with values of 1.62 ± 0.16 and 38.2 ± 1.35%, respectively.

### 3.3. Scanning Electron Microscopy of the Microcapsules

β-cyclodextrin is considered to be the most common spray drying aid protectant, and naked bacteria cannot survive spray powder because of insufficient dry matter content, so the β-cyclodextrin group was taken as the CK control group [30]. The morphologies of the different microcapsules are shown in Figure 2C. The microcapsules had a spherical structure as a whole, with some parts concave inward in the middle. This was because the water in the droplets evaporated rapidly. Because of the high-temperature process, the particles expand to form an outer shell, and the evaporation of water and high pressure inside can cause the small encapsulated balls to rupture [31]. No such rupture phenomenon was observed in the WP-KC group, indicating that the embedding material has low breathability and can provide good embedding protection for the bacterial cells, which is conducive to improving its encapsulation efficiency and the storage stability of the microcapsule in later stages [32]. No bacterial structures were observed on the surfaces of the microcapsules, indicating that *L. paracasei* was completely encased in the wall material. The size range of the microcapsules obtained by spray drying was about 2–10 μm. As shown in CK, *β*-cyclodextrin embedded large broken particles. The surface was not smooth, and there were smaller attached particles, which did not have a good embedding effect on bacteria, and the size was 1.7–11.7 μm. The WP-KC group showed good morphological characteristics from embedding as a whole, with smooth and less obvious depressions, no obvious broken particles, and sizes ranging from 1.8 to 10.0 μm. The WP-XG group showed significant surface cracks ranging in size from 2.1–5.6 μm. The WP-SA group showed relatively small embedded particles with good size uniformity, but the surface suffered from severe dehydration and shrinkage, with significant damage. The overall size ranged from 2.6 to 5.5 μm, with some reaching 8–10 μm. The small particles in the WP-LMP group were nested within the large broken shells, with a diameter range of 2.2–4.6 μm.

### 3.4. Particle Size and Zeta Potential of Microcapsules

**Figure 3:**
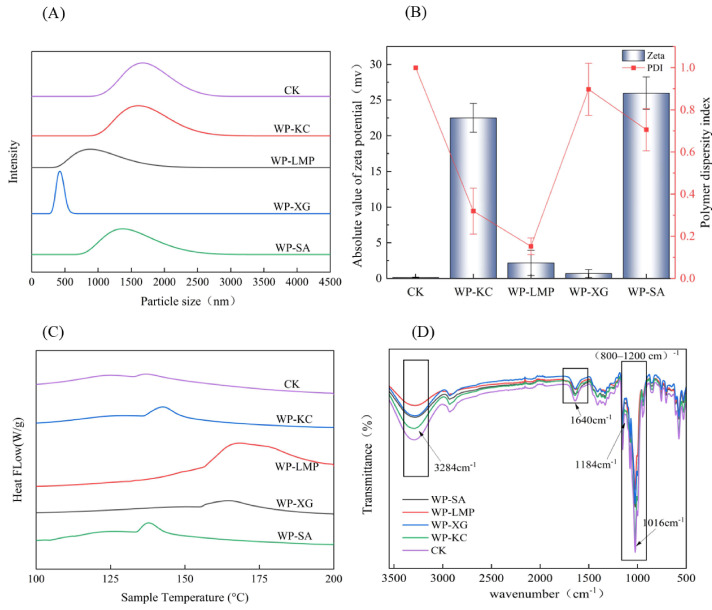
Microcapsule particle size (**A**). Zeta potential diagram (**B**). Microcapsule differential scanning image (**C**). Infrared spectrogram of microcapsule (**D**).

As shown in Figure 3A, the smallest particle size of the microcapsules embedded in the combination of WP-XG is 0.4 μm, followed by WP-LMP microcapsules at 0.8 μm. The particle size of the WP-SA combination was 1.3 μm. After the combination of WP-KC, the particle size is the same as that of the CK group (maltodextrin embedding) at 1.7 μm. Overall, the encapsulated microcapsules reached the micrometer level. This is because the WP-KC complex may have stronger water retention, shrink less during drying, and retain a larger original drop size [33]. A better water-holding capacity corresponded to a higher number of viable bacteria in the powder, which was consistent with the aforementioned longer dissolution time and was conducive to the slow-release type of bacteria.

The zeta potential is an important parameter for measuring the surface charge state of particles. In solution, the surface charge of the particles can affect their dispersibility. Specifically, when particles have a surface charge, electrostatic repulsion is generated between them, which helps to disperse the particles in the solution. The larger the absolute value of the zeta potential, the stronger the electrostatic repulsion between particles, and the less likely the particles are to agglomerate. Therefore, this enhances the dispersibility and stability of the solution [34]. As shown in Figure 3B, the absolute values of the zeta potential in the WP-KC group and the WP-SA group are relatively large, so these two groups have good dispersibility and stability. The smaller the polymer dispersity index (PDI), the more evenly dispersed and stable the particles were in the solution. The PDI value of the WP-KC group is 0.319 ± 0.034 (*p* < 0.01), second only to the WP-LMP group at 0.152 ± 0.021 (*p* < 0.01). Therefore, the overall effect of WP-KC as a microcapsule wall material is the best.

### 3.5. The Differential Scanning Calorimetry of the Microcapsules

As shown in Figure 3C and Table 2, DSC analysis was performed on CK, WP-KC, WP-LMP, WP-XG, and WP-SA. DSC curves showed that the five samples exhibited melting heat absorption at 136.96 °C, 137.94 °C, 164.62 °C, 142.63 °C, and 168.37 °C, respectively. It has been proven that the WP-XG and WP-LMP groups have higher temperature stabilities; however, the wider peak widths of the two groups indicate that the crystalline structure is less orderly [35]. In the remaining groups, WP-KC exhibited high-temperature stability, a narrow peak width, an enthalpy of 9.73 (J/g), and a stable crystal structure.

### 3.6. IR of Microcapsules

As shown in Figure 3D, the WP-KC, WP-SA, WP-XG, and WP-LMP samples exhibited shorter -OH tensile strength at a wavenumber of 3284 cm^−1^ compared to the CK group and a weaker spectrum at a wavenumber of (800–1200) cm^−1^, indicating complex formation between polysaccharides and WP. Absorption bands associated with free carboxyl group (C=O) stretching are shown at 1640 cm^−1^, and those associated with amide II (N-H) bending at 1525 cm^−1^ [36]. At a wavenumber of 1184cm^−1^, the vibration intensity of the C-O-C glycoside bond increased, indicating that the polysaccharide was bound to the protein through hydrogen bonding or hydrophobic interactions, resulting in a closer alignment of sugar chains and the formation of crystalline regions, which limited the free vibration of the glycoside bond and thus enhanced the absorption peak strength. This makes the complex structure denser and enhances the compression resistance of the microcapsule. The ordered structure delays the release of active ingredients. In the infrared spectrum, the absorption peak at 1016 cm^−1^ is usually associated with the stretching vibration of the C-O bond. The peak intensity of WP-KC group was found to be lower than that in the CK group, indicating that the interaction between polysaccharides and proteins was enhanced, and the structure was more stable.

### 3.7. XRD of Microcapsules

**Figure 4:**
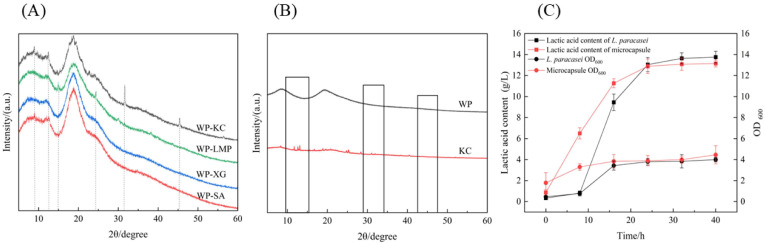
The XRD of four polysaccharide–protein microcapsules (**A**). The XRD of a single sample (**B**). The growth curves and lactic acid content changes in microcapsule powder and *L. paracasei* (**C**).

The diffractograms of crystalline materials have highly ordered structures and display sharply defined peaks, whereas those of amorphous materials show broad scattering peaks due to disordered molecules [37]. To elucidate the crosslinking structure of WP and the four polysaccharides, XRD analysis was performed on the four microcapsule powders, as shown in Figure 4A. WP-SA and WP-XG showed wide peaks at 2θ = 15–20° and did not exhibit obvious sharp and strong crystal peaks, indicating that they were amorphous structures [38]. After mixing WP with KC, the chemical structure may have changed due to interactions. Two sharp crystal peaks appeared at 32° and 45°, which, in turn, affected the position of the diffraction peaks in the XRD patterns [39]. The XRD patterns of single WP and KC showed no crystal structure before crosslinking, as shown in Figure 4B, which proved that new characteristic diffraction peaks were generated after the two were mixed and combined, indicating that the combination of polysaccharides and proteins leads to a change in the molecular arrangement of the resulting mixture.

### 3.8. WP-KC Microcapsule Properties

#### 3.8.1. Storage Stability

Based on the above results, the storage stability of WP-KC was studied. As shown in Table 3, at 45 days, the number of live bacteria in the powder stored at 4 °C was 10.55 ± 0.34 lg CFU/g, while the number of live bacteria in the powder was 10.88 ± 0.20 lg CFU/g when stored at −20 °C and 9.94 ± 0.04 lg CFU/g when stored at 25 °C. Similarly, after 45 days of storage, the survival rate of the powder stored at 4 °C was reduced to 80%, while the survival rate of the powder stored at 25 °C was reduced to almost 0% [40].

Within 60 days of storage, the number of live bacteria in the powder stored at −20 °C and 4 °C was almost the same. At 45 days, the number of live bacteria in the powder stored at 4 °C and −20 °C was 9.91 ± 0.08 lg CFU/g and 9.88 ± 0.03 lg CFU/g, respectively. Meanwhile, the number of live bacteria was 8.81 ± 0.14 lg CFU/g at 25 °C. Finally, after 120 days of storage, the live bacteria number at 4 °C was 8.68 ± 0.03 lg CFU/g (*p* < 0.01), which was higher than 6.34 ± 0.13 lg CFU/g and 4.27 ± 0.05 lg CFU/g at −20 °C and 25 °C. In summary, the bacterial powder had the lowest number of live bacteria when stored at 25 °C, and the optimal storage condition is 4 °C [41].

#### 3.8.2. Growth and Acid-Producing Ability

The growth characteristics and acid-producing capacities of the embedded bacterial powder and the normal activated bacterial solution are shown in Figure 4C; the density of the bacteria tended to be the same after culture for 40 h. Compared to free bacteria, the embedded strains could grow, ferment, and produce acid normally without being affected by encapsulation, and they all showed similar growth patterns. However, the embedded powder showed faster growth and higher strain density when cultured for 0–16 h [42]. The number of viable bacteria peaked at 32 and 40 h, and the UV OD600 of the free bacteria and embedded bacterial powder was stable at 3.99 and 4.45, respectively. In both cases, there were no obvious differences in bacterial growth or lactic acid production.

### 3.9. Stability of Microcapsules in Processing

#### 3.9.1. Thermal Processing

**Figure 5:**
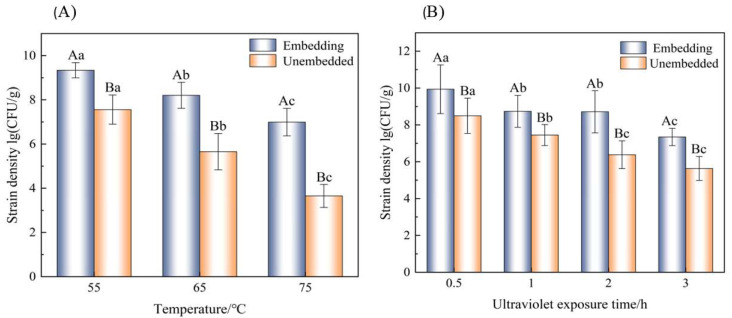
Temperature stability of embedded and unembedded (**A**). UV stability of embedded and unembedded *L. paracei* (**B**). Note: Lowercase letters represent significant differences within the group, denoted by a, b, c. The capital letters represent significant differences between the embedded and unembedded groups, denoted by A, B.

The bacterial densities of the embedded bacteria powder and the unembedded bacteria liquid were treated at 55 °C, 65 °C, and 75 °C for 10 min, as shown in Figure 5A. At all temperatures, the number of live bacteria in the encapsulated bacterial powder was higher than that in the unencapsulated bacterial liquid, which proved that the thermal stability of the bacteria after embedding was improved, and it was more resistant to the extremely high temperature environment encountered during storage and transportation. It was therefore proved that the embedding of κ-carrageenan and whey protein was beneficial to the heat resistance of the strains. In addition, regardless of whether the strains are encapsulated or not, their strain density is affected by temperature.

#### 3.9.2. Ultraviolet Irradiation

Ultraviolet irradiation in food processing is an efficient, non-thermal physical sterilization technology, mainly through the use of short-wave ultraviolet irradiation to destroy the DNA/RNA structure of microorganisms, inhibiting their reproductive ability. However, it can also cause damage to active bacteria [43]. It is particularly important to manage normal ultraviolet sterilization during powder applications. As shown in Figure 5B, with the increasing ultraviolet irradiation time, the number of viable bacteria gradually decreased; however, the number of viable bacteria in the embedded group was always higher than that in the unembedded group. After 3 h of ultraviolet irradiation, the number of viable bacteria in the embedded group gradually decreased to 7.34 log CFU/g, and that in the unembedded group gradually decreased to 5.63 log CFU/g. *L. paracasei* has a stronger resistance to ultraviolet irradiation after embedding.

## 4. Conclusions

The purpose of this study was to evaluate the protective effects of WP-KC, WP-XG, WP-SA, and WP-LMP on the survival of *L. paracasei* F50 after the long-term storage of the microcapsule wall material and to improve the activity of *L. paracasei* F50. Microcapsules were prepared using the spray drying method, the number of viable bacteria was determined, and the powder was characterized. The results showed that WP-KC had superior performance as a wall microcapsule, the highest viable bacteria count was (9.62 ± 0.62 lg CFU/g), and the survival rate was (91.85 ± 2.42%). Its main advantages include low moisture content, low hygroscopicity, and controlled dissolution time. The lower Hausner Ratio (HR) and Carr Index (CI) also revealed excellent powder fluidity, which is beneficial for storage stability, all of which minimizes cell damage during drying. The surface of the WP-KC microcapsules was smooth and without fractures, and the particle size was uniform. After embedding, the microcapsules all had good thermal stability. Among them, the structure of the WP-KC group weakened the stretching of the -OH bond, increasing the vibration intensity of the C-O-C glycosidic bond, and a new crystallization peak appeared, making its structure more compact and stable. After 120 days of storage, the WP-KC group maintained a bacterial density of 8.68 ± 0.03 lg CFU/g at 4 °C, significantly outperforming the others. During thermal processing and ultraviolet irradiation, WP-KC microencapsulation also had an excellent protective effect. In summary, WP-KC was the best encapsulation system for the inclusion of whey protein and the four polysaccharides. Whey protein and carrageenan form a multi-level protection network through the electrostatic interaction of complex condensation, supplemented by hydrogen bonding and hydrophobic interaction, ensuring viability and storage stability of the strains. These findings provide a strategic framework for optimizing probiotic delivery systems in functional foods, highlighting the critical role of κ-carrageenan in enhancing microbial vitality under industrial processing conditions. Based on the excellent controlled release performance and stability of WP-KC microcapsules, its application potential in functional food, drug embedding, and special medical formulations can be explored, especially for probiotic products requiring high-temperature processing or long-term storage at room temperature. It is more likely to lead the innovation of functional food carrier technology, helping the health industry to move forward in the direction of efficiency, stability, and precision.

## Figures and Tables

**Table 1:** The moisture content, solubility, and hygroscopicity of the microcapsules.

Sample	Moisture Content (%)	Solubility (s)	Hygroscopicity (g/100 g)
CK	2.12 ± 0.01 ^b^	900 ± 20.53 ^a^	2.08 ± 0.24 ^a^
WP-KC	2.12 ± 0.01 ^b^	780 ± 13.42 ^b^	1.02 ± 0.35 ^b^
WP-LMP	2.35 ± 0.02 ^a^	540 ± 8.34 ^d^	1.03 ± 0.03 ^b^
WP-XG	2.12 ± 0.01 ^b^	660 ± 12.84 ^c^	1.02 ± 0.52 ^b^
WP-SA	2.12 ± 0.02 ^b^	300 ± 15.36 ^e^	1.02 ± 0.33 ^b^

Note: The letters a, b, c, d and e indicate significant differences in results in the same column.

**Table 2:** DSC analysis of thermal properties and enthalpy change in microcapsules.

Sample	Peak Temperature (°C)	Initial Temperature (°C)	Termination Temperature (°C)	Enthalpy Change (J/g)
CK	136.96 ± 0.85	132.64 ± 0.72	142.36 ± 1.10	30.21 ± 0.43
WP-KC	142.63 ± 1.23	135.90 ± 0.95	149.38 ± 1.65	9.73 ± 0.28
WP-LMP	168.37 ± 2.15	157.23 ± 1.80	196.52 ± 3.40	2.06 ± 0.15
WP-XG	164.62 ± 1.90	155.22 ± 1.45	175.15 ± 2.20	9.86 ± 0.31
WP-SA	137.94 ± 0.78	133.89 ± 0.68	143.11± 1.05	6.82 ± 0.22

**Table 3:** Strain density of microcapsules under different storage temperatures.

Time (Day)	−20 °C (lg CFU/g)	4 °C (lg CFU/g)	25 °C (lg CFU/g)
0	12.41 ± 0.14	12.41 ± 0.14	12.41 ± 0.14
15	12.35 ± 0.06	12.22 ± 0.06	11.59 ± 0.24
30	10.26 ± 0.27	11.94 ± 0.14	10.26 ± 0.05
45	10.88 ± 0.20	10.55 ± 0.34	9.94 ± 0.04
60	9.88 ± 0.03	9.91 ± 0.08	8.81 ± 0.14
75	8.34 ± 0.14	9.98 ± 0.11	8.43 ± 0.23
90	7.32 ± 0.22	9.65 ± 0.11	7.46 ± 0.16
105	6.32 ± 0.35	8.72 ± 0.03	5.46 ± 0.43
120	6.34 ± 0.13	8.68 ± 0.03	4.27 ± 0.05

## Data Availability

The original contributions presented in this study are included in the article. Further inquiries can be directed to the corresponding author.
